# The increasing importance of Dengue virus infection in Saudi Arabia: A review

**DOI:** 10.1016/j.virusres.2024.199510

**Published:** 2024-12-24

**Authors:** Ahmad M. Alharbi

**Affiliations:** Department of Clinical Laboratories Sciences, College of Applied Medical Sciences, Taif University, Taif, 21944, Saudi Arabia

**Keywords:** Dengue virus, Saudi Arabia, Clinical manifestation, Epidemiology, Dengue virus biology

## Abstract

Exacerbated by the rise of global warming due to climate change, as well as ease of international travel and mass migration, the dengue virus infection remains of particular economic and global concern. Of note, the emergence of the first case of dengue viral infection occurred in Saudi Arabia in the 1990s, and since then there has been a steady rise in the number of cases. Moreover, the arrival of imported dengue virus variants poses a significant challenge to dengue fever surveillance and control efforts within the region, especially as Saudi Arabia attracts millions of religious pilgrims throughout the year. Herein, we discuss the epidemiology of dengue viral infection in Saudi Arabia, dengue fever biology and clinical manifestation. Current management strategies, amongst other factors influencing dengue fever in Saudi Arabia are also deliberated upon. Future ongoing research and consistent monitoring of both established and emerging dengue viral strains within Saudi Arabia are needed, given the lack of current comprehensive studies.

## Highlights

• Rising Dengue Incidence: Dengue cases in Saudi Arabia have sharply increased since the 1990s, influenced by climate change and global travel.
• Impact of Pilgrimages and socio-environmental factors are likely contributing to the spread of novel dengue viral strains.
• Emerging research Needs: Ongoing research is essential to address environmental factors in dengue virus transmission.

## 1 Introduction

Arboviruses, the largest known class of viruses, exist in nature through biological transmission between blood-feeding arthropods and their vulnerable vertebrate hosts, or through the transovarial transfer of arthropods (Lessa et al., 2023). Diseases like monkeypox, chikungunya, zika and especially dengue are of considerable epidemiological and clinical concern among them (Araújo et al., 2022). Dengue is the leading arboviral disease among humans, and it is caused by a Flavivirus arbovirus within the Flaviviridae family. It is transmitted through the bites of *Aedes aegypti* or *Aedes albopictus* mosquitoes, and half of the world's population is at risk of contracting the disease (Gubler, 2011; Ferreira-de-Lima and Lima-Camara, 2018). The dengue virus consists of a single-stranded ribonucleic acid (RNA) enclosed in an icosahedral capsid protein coat (Neves-Martins et al., 2021).

**Fig. 1:**
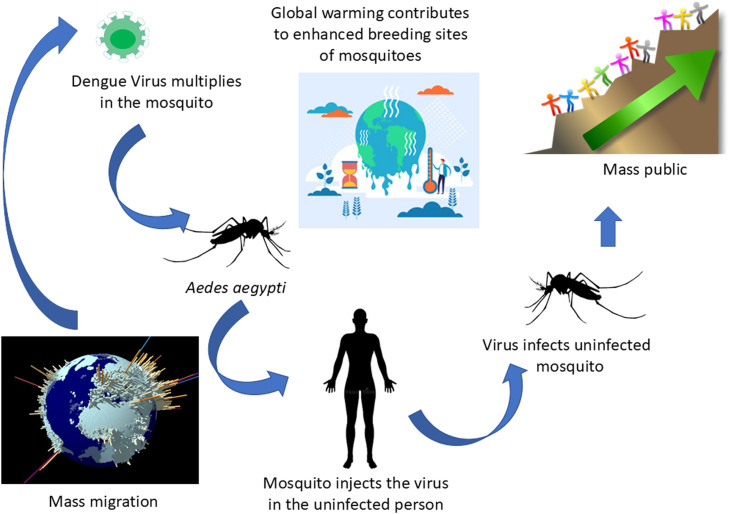
Global warming and mass migration/mass gatherings may contribute to increasing number of dengue cases in Saudi Arabia.

Over the past decade, dengue fever and dengue hemorrhagic fever have re-emerged as serious illnesses that have spread to many different parts of the world (Gubler, 2002). Saudi Arabia, a pivotal nation in the Arab region, holds a crucial role during infectious disease pandemics because millions of Muslim pilgrims annually travel there for mass gatherings, such as religious pilgrimage of Hajj, as well as a large expatriate population, amongst other factors (Fig. 1) (Hoang et al., 2020; Altassan et al., 2019). While the majority of dengue research has concentrated on Latin America and Asia due to their high disease burdens, there is an essential requirement for investigations in regions like Saudi Arabia. In these areas, dengue fever is a public health concern, yet it remains less comprehensively studied and may exhibit unique characteristics. Since the primary case of dengue fever reported in the 1990s, currently Saudi Arabia has one of the largest dengue fever burdens in the Middle East (Altassan et al., 2019). Herein, we detail the epidemiology and impact on Saudi Arabia owing to the emergence of dengue virus in the 1990s. Furthermore, we also consider the role of climate change, increased globalization amongst a plethora of factors, such as interaction with other environmental pathogens.

## 2 Epidemiology of dengue with a focus on Saudi Arabia

The World Health Organisation (WHO) considers dengue as a serious illness that poses substantial risks to the health of the global populace given the recent sharp rise in cases and deaths that are related with the disease (Zeng et al., 2021). According to projections, more than 6 billion individuals may be endangered by dengue in 2080, more than twice as many cases as there were in 2015 (Messina et al., 2019). Dengue is estimated to afflict at least 4 billion people around the globe, or 50 % of the global population, with an annual infection rate of 400 million infections and a symptom prevalence of 50 to 100 million (Messina et al., 2019). According to these statistics, dengue is the most common arbovirus in the world and is primarily found in tropical and subtropical regions as well as the Pacific, Southeast Asia, and the Americas, endangering the health of over 2.5 billion individuals. Globalisation and increased global travel, growth of population, and urbanisation are some of the factors that have been linked to the resurgence of dengue (Kolimenakis et al., 2021). There are four primary genetically related serotypes of the virus including: DENV-1, DENV-2, DENV-3, and DENV-4 coexisting amongst humans, but are distinct antigenically (Lee et al., 2023). A fifth serotype (DENV-5) was identified in Malaysia, Asia, in 2013 (Mustafa et al., 2015). Amidst the various dengue virus serotypes, DENV-1 is considered as most virulent, with a capability to trigger significant epidemics within a short timeframe, nonetheless, this necessitates further research as both DENV-2 and DENV-3 may also display considerable virulence under certain conditions. The virulence of dengue virus serotypes is a complex issue influenced by various factors, such as the host's immune response and the co-circulation of multiple serotypes in an area (Lessa et al., 2023).

Historical records document cases of diseases resembling dengue in the Arabian region during the 19th and early 20th centuries (El-Kafrawy et al., 2016). Following a period of several decades without any reports of dengue fever, the illness has resurfaced in the Middle East and North Africa (MENA) region. Saudi Arabia remained dengue-free until the mid-1990s (Shepard et al., 2016). However, starting in 1994, there have been numerous outbreaks, and the number of cases has been steadily increasing, with 6512 cases reported in 2013 and approximately 3000 in 2019 (Al-Tawfiq and Memish, 2018). The majority of cases occurred in Jeddah, followed by Jazan and Makkah regions (Zaki et al., 2008). Previous research has documented the presence of all DENV serotypes in various regions, with DENV-2 being the most prevalent, followed by DENV-1, as revealed by a cross-sectional study involving the collection of serum samples from 910 eligible male blood donors in Saudi Arabia, between 2015 and 2016, and serum samples from 220 suspected dengue cases, in 2016 (ALSHEIKH et al., 2017; Ashshi, 2017). Since 2005, the Jazan region in southwestern Saudi Arabia has experienced multiple dengue virus outbreaks (ALSHEIKH et al., 2017; Ashshi, 2017). Notably, the Jazan region experienced a large increase in dengue virus cases in 2019 and 2020 (Alshabi et al., 2022). From 2006 through 2020, there were almost 4600 dengue cases reported, with the peak occurring in 2019 and 876 cases reported in 2020 (Alshabi et al., 2022). Recent research has shown that there are three serotypes of the dengue virus present in the area, namely DENV-1, DENV-2, and DENV-3, with DENV-2 being the most prevalent serotype (Alshabi et al., 2022; Dafalla et al., 2021; Mohammed et al., 2018).

Over time, sporadic outbreaks occurred, with Jeddah experiencing small outbreaks, each consisting of no more than 15 reported cases Altassan et al. (2019). The disease expanded to more cities in the region between 2004 and 2015, leading the Saudi Ministry of Health to define the area as dengue fever endemic Altassan et al. (2019). In 2015, the incidence of dengue fever was recorded at 13.68 cases per 100,000 individuals Altassan et al. (2019). Males between the ages of 15 and 30 make up the majority of these cases, primarily because they are more inclined to work in outdoor occupations like farming and shepherding. In addition, women generally wear covered clothing due to cultural norms, which likely reduces their exposure to mosquito bites.

A previous study examined the phylogeny of 81 dengue virus isolates collected in Jeddah, Saudi Arabia, from 1994 to 2006 Zaki et al. (2008). This analysis included 37 DENV-1 isolates, 32 DENV-2 isolates, and 12 DENV-3 isolates, with no DENV-4 detected. The findings indicated that DENV-1 had likely been introduced into Saudi Arabia on at least two separate occasions. The study noted that during the initial 1994 outbreak, DENV-1 and DENV-2 co-circulated, starting in the summer and coinciding with the Haj season, with both serotypes persisting throughout the year. It was not until 2004, a decade later, that DENV-1 and DENV-2 re-emerged, with DENV-2 being the predominant serotype. The 2004 DENV-2 isolates belonged to the same Cosmopolitan genotype observed in 1994, while the DENV-1 strains in 2004 were of the Asia genotype, distinct from the America-Africa genotype seen in 1994. This Asia genotype of DENV-1, though initially a minor presence in 2004, continued to circulate and triggered a prolonged outbreak from the summer of 2005 until early 2006 (Zaki et al., 2008).

Dengue importation and potential outbreaks pose significant public health challenges during mass gatherings such as Hajj and Umrah in Saudi Arabia and various factors that may influence dengue fever in Saudi Arabia are displayed in Table 1. A comprehensive, multi-layered approach is essential to effectively mitigate these risks. Enhanced surveillance and monitoring should be prioritized, including rigorous vector surveillance for *Aedes* mosquitoes in key pilgrimage areas and real-time genomic sequencing of dengue virus strains to detect new serotype introductions early. Public health preparedness measures such as health screenings for incoming pilgrims from dengue-endemic regions and swift isolation protocols for suspected cases are crucial for limiting local transmission. Vector control programs should be intensified, incorporating targeted insecticide spraying, larvicide treatments in stagnant water, and innovative solutions like the release of Wolbachia-infected mosquitoes to sustainably curb vector populations. Furthermore, coordinated travel advisories and pre-travel education could inform pilgrims about dengue risks and encourage preventive behaviour. By integrating these measures, Saudi Arabia may further enhance its public health response, protecting both residents and visitors during major religious events from the risk of dengue importation and outbreaks.

**Table 1:** Factors that may influence dengue fever in Saudi Arabia.

Factors	Influence on dengue fever
Availability of water and practices related to water storage. Altassan et al. (2019)	Potential breeding sites for mosquito vectors
Electricity availability in households. El-Gilany et al. (2010)	May effect mosquito control and preventative measures
Urban expansion and advancement of urban areas. Altassan et al. (2019)	Alters mosquito habitats and increases exposure
Geographic proximity to regions where Increased risk of dengue fever is prevalent (e.g., Yemen). Altassan et al. (2019)	Increased of risk of introduction of the virus travel
Well established travel networks. El-Gilany et al. (2010)	Facilitates movement of infected individuals
Demographic and social shifts, including population density, housing, water supply, sanitation and waste management. Zaki et al. (2008); Alswaidi et al. (2013)^.^	Contributes to conditions conducive to transmission of vector borne diseases
Movement of people within the population Introduction of new viral strains (e.g., foreign labourers and religious pilgrims). Altassan et al. (2019); Zaki et al. (2008); Alswaidi et al. (2013)	Introduction of new viral strains
Large-scale gatherings during Hajj and Umrah pilgrimages (Hoang et al., 2020).	Potential introduction of dengue virus strains and emergence of new strains.
Possibility of introducing the dengue virus through Saudi citizens traveling to regions where dengue fever is endemic regions during travel for business and leisure purposes. Zaki et al. (2008); Alswaidi et al. (2013)	Potential introduction of dengue virus strains and emergence of new strains

## 3 Dengue fever biology and clinical manifestation

Human infection with the dengue virus starts after a bite from an *Aedes* mosquito carrying the virus. Although the spleen, liver, kidney, and lymph nodes are among the tissues and organs in which the dengue virus can proliferate, monocytes, dendritic cells, and macrophages are the virus's primary targets. The dengue virus life cycle involves numerous stages, comprising of viral entry, replication, assembly, and eventual virion release. Initially, dengue virus particles bind to host cells by engaging with receptors on the host cell and surface proteins on the virus. Mannose receptors, the human C-type lectin-like molecule, the dendritic cell-specific intracellular adhesion molecule-3-grabbing non-integrin, and other host receptors are thought to be involved in this interaction (Huerta et al., 2008). According to recent research, the third domain of the dengue virus's E protein may be extremely important to recognise receptors and is essential for facilitating the entry of the dengue virus into host cells, via processes such as receptor-mediated or clathrin-dependent endocytosis Swaminathan and Khanna (2009). Additionally, the fusion of the host cell membrane and the dengue virus may allow the virus to directly enter host cells (Krishnan et al., 2007).

The acidic environment of the endosome that the dengue virus encounters after internalisation into the host cell leads to a series of events. As a result, domain II of the E protein dimers moves away from the surface of the virus, leading the E protein dimers to separate. As a result, the fusion loop enters the endosomal membrane, connecting the viral and endosomal membranes, and starting the domain rearrangements that lead to the production of E protein trimers. The E protein's domain III rotates and forms connections with other domains throughout this process, ultimately leading to pore formation which enable release of the dengue viral genome into cell cytoplasm of the host cells. Subsequently, within the host cytoplasm, the viral RNA is translated and cleaved by both viral and host proteases to generate dengue structural and non-structural proteins, which are vital for replication of the virus. The site of virion assembly is the endoplasmic reticulum (ER), where the ER membrane and viral glycoproteins enclose the C proteins and newly synthesized viral RNA into immature dengue viral particles. These developing particles traverse along the trans-golgi network and secretory route. Here, host furin proteases process the prM protein to form the mature M protein. Finally, after effective maturation, the viral particles are exocytosed from host cells, enabling them to infect new host cells in a continuous cycle of infection and replication (Lee et al., 2023; Klein et al., 2013; Liao et al., 2010).

Approximately 80 % of initial dengue virus infections occur without any noticeable symptoms, with fewer than 20 % of those infected showing clinical signs. Dengue fever typically manifests as intense headaches, mild fever, skin rashes, muscle and joint discomfort, as well as feelings of nausea and vomiting Khan et al. (2023). Dengue hemorrhagic fever is marked by elevated body temperature, enlarged liver (megalohepatia), instances of bleeding, shock, and frequently, disruptions in cardiovascular function. Initially, DHF was documented as primarily impacting individuals under the age of 15, but subsequent research has revealed its occurrence in adults as well (Khan et al., 2023).

Infection with the dengue virus initiates acute febrile syndrome Khan et al. (2023); Qureshi et al. (1997). Some studies suggest that the dengue virus non-structural protein-1 is found abundantly in a patient's serum, both as a soluble lipoprotein and on cell surfaces, and these elevated levels might be linked to disease severity and play a role in the development of severe dengue in the host McBride (2009); Datta and Wattal (2010); Yow et al. (2021). A single infection with a specific dengue virus serotype can grant long-lasting immunity against that serotype and offer limited cross-protection against different serotypes in the short term. However, it has been observed that a second infection with a different dengue serotype, known as heterotypic secondary dengue viral infection, significantly increases the risk of severe dengue, including the potentially life-threatening conditions of dengue hemorrhagic fever and dengue shock syndrome Kuo et al. (2018); Wang et al. (2020). Moreover, certain clinical symptoms and non-communicable conditions such as high blood pressure and diabetes have been recently linked to the severity of dengue infection (Pang et al., 2012; Singh et al., 2022; Lye et al., 2016).

## 4 Current management strategies

Despite dengue infection being prevalent worldwide, there is presently no effective antiviral therapy available, and the conventional treatment approach continues to focus on providing supportive care Chen (2023). Hence, there is an urgent demand for antiviral medications with the potential to effectively treat severe dengue infections Low et al. (2021). Since there is no specific recommended treatment for dengue, the primary approach is to provide supportive care. This typically involves fluid replacement therapy, with 0.9 % saline as the initial choice, administered through either infusion or bolus dose in cases of shock. This approach is backed by evidence to prevent hypovolemia and the occurrence of any complications. While acetaminophen can be used to reduce fever, it's important to avoid nonsteroidal anti-inflammatory drugs, as they can raise the risk of bleeding. Whether hospitalization is necessary depends on the patient's condition, and they should be carefully assessed and closely monitored for any concerning signs (Rajapakse et al., 2012).

Creating a safe and effective dengue vaccine has proven immensely challenging, despite being a vital element in the multifaceted strategy to combat dengue on a global scale. Over the past 75 years, scientists and developers have made significant efforts to design such vaccines, but they have faced formidable challenges (Thomas, 2023). While various approaches have been under investigation, only vaccines using live attenuated viruses have progressed to licensure or advanced stages of clinical development Thomas (2023). At this time, only Denvaxia (Sanofi Pasteur Inc. USA) has been permitted licensing, as well as Takeda: TAK-003 Percio et al. (2024). The initial findings from the long-term safety study of Dengvaxia trials showed promising efficacy in reducing hospitalization, but this effectiveness is significantly influenced by the individual's prior exposure to dengue virus and their age at vaccination Percio et al. (2024). There is a need for more research to uncover the reasons behind the reduced efficacy in seronegative individuals and the increased risk of hospitalization in children under 9 years old. These questions remain unresolved and are the subject of ongoing investigation Percio et al. (2024). Vaccination for dengue and programs aimed at controlling the spread of the disease could provide a cost-efficient strategy, and could be explored as a strategy in Saudi Arabia (Akbar et al., 2020).

Controlling disease vectors is crucial in preventing dengue fever Wilson et al. (2020). This involves actions like using larvicides and adulticides, preventing mosquito breeding by covering standing water, utilizing chemical agents with active substances like N,N-Diethyl-meta-toluamide and picaridin to safeguard water sources, employing insect repellents, wearing long clothing treated with permethrin as an insecticide, and adopting various other measures. Additionally, it's important to secure homes by covering windows and implementing mosquito prevention measures like mosquito nets. Educational programs about disease transmission and preventive measures should also be promoted, along with the establishment of surveillance programs to monitor disease prevalence Bowman et al. (2016). Crucially, enhancing efforts to control disease-carrying vectors should be a vital element of healthcare planning in Saudi Arabia. This is especially pertinent given the large-scale gatherings during Hajj and Umrah, which predominantly involve people from Southeast and South Asia. There is a risk of these events facilitating the transmission of dengue and other neglected tropical diseases carried by vectors (Almutairi et al., 2018).

## 5 Factors influencing dengue fever in Saudi Arabia

Several socio-environmental factors may play a role in the occurrence and dissemination of dengue fever in Saudi Arabia and are summarised in Table 1 Altassan et al. (2019). These factors encompass aspects such as the availability of water and the practices related to water storage, electricity available in households, the swift expansion and advancement of urban areas, the close geographical proximity to regions where dengue fever is prevalent (Yemen), and the well-established transportation networks Altassan et al. (2019). Throughout the nation, demographic and social shifts, including factors like population density, housing, water supply, and inadequate sanitation and waste management systems, may be contributing to conditions that could be conducive to heightened transmission of mosquito-borne diseases and other vector-borne illnesses El-Gilany et al. (2010).

The movement of people within the population is another significant factor influencing the transmission of dengue fever through the introduction of new strains of the dengue virus. Primary sources of this introduction include foreign labourers and religious pilgrims. A phylogenetic analysis indicated that the virus was probably brought into the country by expatriate workforce, religious pilgrims, or Saudi citizens traveling overseas Zaki et al. (2008). As already stated, Saudi Arabia depends on a substantial expatriate labour force, primarily hailing from the Indian subcontinent and Southeast Asia. This workforce presently constitutes approximately 30 % of the population Alswaidi et al. (2013). As many of this workforce may originate from dengue-endemic regions, they may have been the initial source of dengue virus, as well as a possible source of importation of strains. Furthermore, the possibility of introducing the dengue virus (DENV) exists through Saudi citizens traveling to regions where dengue fever is endemic for business and leisure purposes Azhar et al. (2015). Jeddah, where the initial outbreaks occurred, hosts a sizable and culturally diverse population from various parts of the globe, primarily because of its close proximity to the holy city of Makkah. Traditionally, individuals came to Jeddah as pilgrims and later established residence in the area Jamjoom et al. (2016). Moreover, Jeddah is one of Saudi Arabia's principal hubs for commerce and trade, consequently, it could potentially serve as a focal point for the transmission of diseases, particularly to nearby cities like Makkah, and Al-Madinah (Alhaeli et al., 2016).

Muslim pilgrims in Saudi Arabia, especially in Makkah and Al-Madinah, represent a distinctive and significant potential source for the introduction of dengue. These cities are the location of sacred mosques visited by millions of Muslims annually during the Hajj and Umrah pilgrimages. The Hajj, which occurs annually over five days, attracts around 2 million Muslim pilgrims from around the globe, rendering it the greatest mass gathering globally (Alharbi, 2023; Ahmed and Memish, 2020).

Furthermore, the Umrah pilgrimage, which is not bound by a specific time, draws an additional 5 to 6 million Muslim visitors each year Alharbi (2023); Ahmed and Memish (2020). Of note, a substantial portion of these visitors originates from the Indian subcontinent, Southeast Asia, and Eastern Africa, regions where many vector-borne diseases, including dengue fever, are endemic. The phylogenetic examination of dengue virus in Saudi Arabia reveals a strong resemblance to strains found in countries that receive a significant number of pilgrims, such as Pakistan, Indonesia, and India. This implies that pilgrims might be a potential source of dengue virus introduction Hashem et al. (2018). This is corroborated by the fact that the initial outbreak in 1994 coincided with the Hajj pilgrimage Fakeeh and Zaki (2001). Dengue fever cases are typically observed to be clustered in cities visited by pilgrims, notably Jeddah, near the international airport. Pilgrims from eastern Africa likely introduced the Asian genotype of DENV-1 in Saudi Arabia. Multiple introductions of the same strain were evident in DENV-2 and DENV-3, suggesting back-and-forth exchange across countries Altassan et al. (2019). Large-scale gatherings, such as the Hajj and Umrah pilgrimage likely enabled various DENV strains from different regions to come together, and of concern, may potentially lead to the emergence of new viral strains, in future. To enhance the analysis of dengue importation in Saudi Arabia, it will be important to explore how these factors may contribute to the risk of outbreaks. This will involve examining the epidemiological context of dengue in countries with high travel rates, particularly during significant events like Hajj and Umrah. Analysing travel patterns, local environmental conditions, and the potential presence of disease vectors will provide a clearer understanding of the dynamics at play.

To effectively mitigate the risks associated with dengue importation, several strategies may be implemented. Strengthening surveillance systems to monitor dengue cases among travellers is crucial. Public awareness campaigns aimed at educating both residents and visitors about dengue prevention measures will also be beneficial. Additionally, vector control measures should be prioritized in areas with high foot traffic, especially during peak seasons. Health protocols at entry points should be reinforced to facilitate the monitoring of symptoms and provide immediate care when necessary. Furthermore, it is vital to extend educational efforts to travellers from regions in Southeast Asia (India, Pakistan, Bangladesh, Indonesia etc.), ensuring they are informed about dengue risks and prevention strategies. Collaborating with regional health organizations will further bolster public health initiatives, allowing Saudi Arabia to effectively address the challenges posed by dengue importation and reduce the likelihood of outbreaks, in future. Furthermore, implementing a combination of pre- and post-travel strategies in collaboration with countries sending large numbers of pilgrims could significantly enhance dengue prevention efforts. Pre-travel efforts could include screening for dengue symptoms and educating pilgrims on preventive measures, such as using mosquito repellents and wearing protective clothing. Post-travel strategies focus on monitoring returning pilgrims in their home countries, enabling early detection and containment of dengue cases. These measures could significantly reduce the risk of introducing dengue to non-endemic regions while maintaining global health security.

## 6 Concluding remarks

Dengue fever has emerged as a major global health issue, increasingly driven by climate change and other socio-environmental factors as depicted in Fig. 1. A recent study conducted between 2013 and 2017 in Saudi Arabia estimated the economic impact of dengue fever Akbar et al. (2020). The analysis used national case data and a validated predictive model. It considered both direct costs for a total estimated cost in 2016 exceeding $168.5 million, involving over 4000 confirmed cases Akbar et al. (2020). Over the five-year period, the total cost of dengue fever in Saudi Arabia was estimated at $551.0 million, affecting 15,369 patients out of approximately 25,000 reported cases, with an average per-patient cost of $12,000 Akbar et al. (2020). Addressing dengue in Saudi Arabia requires a comprehensive approach, including enhanced surveillance, targeted interventions, and ongoing investigation into the disease's dynamics. With the potential for increased outbreaks due to climate shifts and international travel, prioritizing research and public health initiatives will be crucial in mitigating the impact of dengue fever.

## 7 Future perspectives

The emergence and persistence of dengue fever in Saudi Arabia are influenced by several unique factors. The influx of expatriates and pilgrims from dengue-endemic regions in the Middle East and Asia exacerbating the situation, highlighting the need for targeted vaccine development and robust surveillance to manage the introduction of new dengue virus variants. In addition, the appearance of imported variants of the dengue virus presents a significant obstacle to the surveillance and control efforts for dengue fever. Consequently, it is imperative to conduct ongoing research and maintain continuous monitoring of both existing and newly emerging viral strains in the region.

Furthermore, the influence of climate change, should inform further research, guide preventive measures, and enhance the readiness of the healthcare system to address this public health challenge. Alterations in environmental conditions are having an impact on the geographic spread of infectious diseases. For instance, in the South American region where dengue is prevalent, there's a substantial disease burden and frequent outbreaks. The climate conditions suitable for dengue transmission have reached their peak levels in recent years, with a notable 35.3 % increase during the period from 2012 to 2021 compared to the baseline from 1951 to 1960, thus it is likely, that other regions such as Saudi Arabia may see a rise in dengue infection cases. Furthermore, the current distribution of *Aedes aegypti*, the primary vector for dengue in Saudi Arabia, is concentrated in urban and semi-urban areas, particularly in cities like Jeddah, Makkah, and surrounding regions. The mosquito thrives in water containers and stagnant water near human settlements, making densely populated areas more vulnerable. Factors like climate change, urbanization, and limited access to water management exacerbate its spread. Efforts are ongoing to improve vector control and reduce dengue transmission risks in the country (Nature Middle East 2019).

In conclusion, mitigating the risks of dengue importation during large-scale events such as Hajj and Umrah demands a comprehensive, coordinated approach. Saudi Arabia's role as host to millions of international pilgrims provides both a challenge and an opportunity to strengthen global health measures. By enhancing surveillance systems, adopting innovative vector control strategies, and improving public health preparedness, the country may significantly reduce the threat of dengue outbreaks. Furthermore, fostering partnerships with dengue-endemic countries for data exchange and collaborative research may amplify preventive efforts. Education and awareness campaigns tailored for pilgrims, alongside reinforced healthcare infrastructure, will be essential for rapid identification and response to cases. Integrating vaccination as part of dengue prevention during Hajj and Umrah could be beneficial, particularly for high-risk populations from endemic areas. However, due to the potential risks of the dengue vaccine, it should be recommended that eligible individuals, particularly those with a history of prior dengue infection, consult healthcare providers before receiving the vaccine. This cautious approach, combined with other preventive strategies, could help reduce disease transmission while ensuring the safety of pilgrims. Utilising digital tools, such as Geographic Information System mapping and mobile health applications, could facilitate real-time monitoring of mosquito populations and provide timely health alerts to pilgrims. Enhanced public health education campaigns should emphasize the use of repellents, protective clothing, and the importance of avoiding mosquito breeding sites. Furthermore, addressing climate variability through adaptive vector control strategies and investing in sustainable infrastructure improvements around pilgrimage sites should yield lasting benefits. Together, these measures ensure a robust, multi-layered approach to dengue mitigation. These strategies will ensure the safety of residents and visitors but also position Saudi Arabia as a leader in public health readiness for mass gatherings, offering valuable lessons for other nations.

## Consent for publication

Not applicable.

## CRediT authorship contribution statement

**Ahmad M. Alharbi:** Writing – review & editing, Writing – original draft, Visualization, Validation, Project administration, Investigation.

## Declaration of competing interest

The authors declare that they have no known competing financial interests or personal relationships that could have appeared to influence the work reported in this paper.

## Data Availability

No data was used for the research described in the article.
